# Danish Whole-Genome-Sequenced *Candida albicans* and *Candida glabrata* Samples Fit into Globally Prevalent Clades

**DOI:** 10.3390/jof7110962

**Published:** 2021-11-12

**Authors:** Judit Szarvas, Ana Rita Rebelo, Valeria Bortolaia, Pimlapas Leekitcharoenphon, Dennis Schrøder Hansen, Hans Linde Nielsen, Niels Nørskov-Lauritsen, Michael Kemp, Bent Løwe Røder, Niels Frimodt-Møller, Turid Snekloth Søndergaard, John Eugenio Coia, Claus Østergaard, Henrik Westh, Frank Møller Aarestrup

**Affiliations:** 1 Division for Global Surveillance, National Food Institute, Technical University of Denmark, 2800 Kongens Lyngby, Denmark; anrire@food.dtu.dk (A.R.R.); valeriabortolaia@gmail.com (V.B.); pile@food.dtu.dk (P.L.); fmaa@food.dtu.dk (F.M.A.); 2 Department of Clinical Microbiology, Herlev Hospital, 2730 Herlev, Denmark; dennis.schroeder.hansen.01@regionh.dk; 3 Department of Clinical Microbiology, Aalborg University Hospital, 9100 Aalborg, Denmark; halin@rn.dk; 4 Department of Clinical Microbiology, Aarhus University Hospital, 8200 Aarhus, Denmark; nielnoer@rm.dk; 5 Department of Clinical Microbiology, Odense University Hospital, 5000 Odense, Denmark; mkemp@health.sdu.dk; 6 Department of Clinical Microbiology, Slagelse Hospital, 4200 Slagelse, Denmark; blro@regionsjaelland.dk; 7 Department of Clinical Microbiology, Rigshospitalet, 2100 København, Denmark; niels.frimodt-moeller@regionh.dk; 8 Department of Clinical Microbiology, Hospital of Southern Jutland, 6400 Sønderborg, Denmark; Turid.Snekloth.Sondergaard@rsyd.dk; 9 Department of Clinical Microbiology, Sydvestjysk Hospital, 6700 Esbjerg, Denmark; John.Eugenio.Coia@rsyd.dk; 10 Department of Clinical Microbiology, Vejle Hospital, 7100 Vejle, Denmark; claus.ostergaard@rsyd.dk; 11 Department of Clinical Microbiology, Hvidovre Hospital, 2650 Hvidovre, Denmark; Henrik.Torkil.Westh@regionh.dk; 12 Department of Clinical Medicine, University of Copenhagen, 2200 Copenhagen, Denmark

**Keywords:** whole-genome sequencing, fungal infection, antifungal susceptibility, phylogenetics

## Abstract

*Candida albicans* and *Candida glabrata* are opportunistic fungal pathogens with increasing incidence worldwide and higher-than-expected prevalence in Denmark. We whole-genome sequenced yeast isolates collected from Danish Clinical Microbiology Laboratories to obtain an overview of the *Candida* population in the country. The majority of the 30 *C. albicans* isolates were found to belong to three globally prevalent clades, and, with one exception, the remaining isolates were also predicted to cluster with samples from other geographical locations. Similarly, most of the eight *C. glabrata* isolates were predicted to be prevalent subtypes. Antifungal susceptibility testing proved all *C. albicans* isolates to be susceptible to both azoles and echinocandins. Two *C. glabrata* isolates presented azole-resistant phenotypes, yet all were susceptible to echinocandins. There is no indication of causality between population structure and resistance phenotypes for either species.

## 1. Introduction

Opportunistic fungal infections are an increasing concern in hospital settings, as invasive infections and fungemia have a high rate of mortality. A majority of these infections are caused by *Candida* species, and the incidence of invasive candidiasis has been rising worldwide in recent decades [1]. Denmark is not an exception to this, and although the incidence rate has stabilized at around 8/100,000 inhabitants, it remains higher than in other Nordic countries [2]. Moreover, between 2012 and 2018, an increasing trend could be observed in the level of antimicrobial resistance towards azoles and echinocandins, making the treatment of candidiasis difficult [2,3,4].

*Candida* species normally colonize the skin, intestines, or mucosal surfaces, but may cause disseminated infections in immunocompromised individuals [5]. The most common species causing candidemia is *Candida albicans*, but the species’ composition shows a changing pattern, with *Candida glabrata* and *Candida parapsilosis* infections becoming more prevalent [1,4,6,7].

Currently, the main antifungal agents used to treat infections caused by *Candida* species are azoles (such as ketoconazole, fluconazole, and itraconazole, as well as more recent compounds such as voriconazole and posaconazole) [8]; echinocandins (in particular caspofungin, micafungin, and anidulafungin) [9]; and amphotericin B [10]. Several *Candida* species, notably, *C. albicans* and *C. glabrata,* are able to acquire resistance to these drugs, especially through mutations in the genes coding for the drugs’ targets or enzymes involved in their syntheses, or through the over-expression of efflux pumps. Point-mutations in *FKS* genes that encode the β-1,3-glucan synthase, which is the target for echinocandins, decrease susceptibility towards these antifungals [11,12,13]. Several mutations have also been identified in genes involved in lanosterol 14α-demethylase synthesis, leading to resistance to azoles [14,15,16,17]. Such mutations occur, for example, in the *ERG11* gene, leading to reduced binding of the drugs to their target, or in promotors or transcription factors, causing over-expression of the gene. Over-expression of efflux pumps is another known common mechanism conferring azole resistance: mutations in transcription factors, such as *TAC1*, lead to the over-expression of ATP-binding cassette transporters such as the ones coded by *CDR1* and *CDR2* genes.

*C. albicans* is a predominantly diploid organism that has a highly clonal population structure. Multilocus sequence typing (MLST) has shown that *C. albicans* samples belong to a limited number of major clades with some degree of geographical enrichment [18,19,20]. MLST has the limitation of relying on only seven gene fragments, situated on five chromosomes, which constrains the detection of possible recombination events stemming from the parasexual cycle of *C. albicans* [21,22].

*C. glabrata,* on the other hand, is thought to be an obligate haploid, and sexual reproduction has not been observed in this species, although genetic analysis indicates the capability for it [23,24]. Six locus-based MLST studies have demonstrated clonal populations and clades that are, similarly to *C. albicans*, enriched in certain geographical areas [25,26,27].

Recently, whole-genome sequencing has become available for routine studies with fungal pathogens. It has the advantage over MLST to give increased resolution and information about structural variants that could offer new insights into the mechanisms of virulence. So far, only a limited number of studies have investigated the phylogeny of *Candida* species and have demonstrated that both organisms are capable of recombination and have shown a lack of geographical signal in the phylogenies [22,24,28,29].

The present study was conducted to obtain a snapshot of the diversity of *Candida* species across all 11 Danish Clinical Microbiology Laboratories (DCMs). In addition, the genomes were compared with publicly available genomes from other geographic locations. We show that Danish isolates fit into several global clades, both for *C. albicans* and *C. glabrata*.

## 2. Materials and Methods

### 2.1. Clinical Isolates

On January 10, 2018, bacterial and yeast isolates from routine analyses (*n* = 2073) were collected from all 11 DCMs (Rebelo et al. Submitted); 51 yeast isolates were identified among this collection. Geographical provenance of samples was anonymized by attributing a code (F1 to F11) to each of the 11 DCMs. Metadata for the isolates included in the study are in Supplementary Data 1. The project was approved by the Danish Data Protection Agency, and material transfer agreements were signed between the Technical University of Denmark and all DCMs.

### 2.2. Antifungal Susceptibility Testing

Antifungal susceptibility testing (AST) was performed by broth microdilution using SensititrE™ YeastOne™ YO10 panel (Thermo Fisher Scientific Inc., Waltham, MA, USA) according to manufacturer’s instructions. The control strain *Candida parapsilosis* ATCC 22019 was used. Minimum Inhibitory Concentration (MIC) interpretation was performed according to the European Committee on Antimicrobial Susceptibility Testing (EUCAST) clinical breakpoints (EUCAST Antifungal Clinical Breakpoint Table v10.0) or, where clinical breakpoints were not available, according to the EUCAST epidemiological cut-off values (ECOFFs). The applied thresholds are in Supplementary Data 2.

### 2.3. Whole-Genome-Sequencing Data and Species Identification

Genomic DNA was extracted from the 51 yeast isolates using the Easy-DNA^TM^ Kit (Invitrogen, Carlsbad, CA, USA), and DNA concentrations were determined using the Qubit^TM^ dsDNA high-sensitivity (HS) and/or broad-range (BR) assay kits (Invitrogen, Carlsbad, CA, USA). The genomic DNA was prepared for Illumina pair-end sequencing using the Illumina (Illumina, Inc., San Diego, CA, USA) NexteraXT^®^ DNA Library Prep Reference Guide (Document #15031942, v03, February 2018) and NextSeq System Denature and Dilute Libraries Guide (Document #15048776, v03, April 2018). The libraries were sequenced using the Illumina NextSeq 500 platform. 

Quality control was performed on the sequencing reads with FastQC v.0.11.8 (http://www.bioinformatics.babraham.ac.uk/projects/fastqc/ (accessed on 3 June 2019)), and summarized with MultiQC [30]. The raw reads were trimmed with BBDuk2 from BBMaps v36.49 [31] and assembled with SPAdes v3.11.0 [32] into draft genomes. In silico species identification was performed using KmerFinder v3.1 [33,34,35] on the draft genomes. Only the *C. albicans* and *C. glabrata* isolates were included in this analysis.

### 2.4. Variant Calling and Phylogenetic Analysis

The pipeline for variant calling was constructed using Snakemake [36]. Scripts are publicly available at https://bitbucket.org/jszarvas/candida_phylogeny_pipeline/ (accessed on 19 August 2020).

First, trimmed reads were aligned against the reference genome of *C. albicans* SC5314 or *C. glabrata* CBS138 with minimap2 v2.6 [37]. BAM files were sorted by coordinates with SAMtools v1.6 [38], then duplicated reads were removed with sambamba v0.6.5 [39]. Genome coverage was calculated for each sample, and samples with less than 20× over 80% of the total reference length were excluded from the analysis due to low coverage.

Raw variants were called with the Genome Analysis Toolkit (gatk) v.4.1.3.0 [40] HaplotypeCaller with a call confidence threshold of 10.0. Single-nucleotide polymorphisms (SNPs) and insertions–deletions (indels) were separated by gatk SelecVariants for the purpose of hard filtering with VariantFiltration. SNPs were selected, if they cleared the following filters: QD < 2.0, FS > 60.0, SOR > 3.0, MQ < 40.0, MQRankSum < −10.0, while indels were filtered with the following: QD < 2.0, FS > 200.0, SOR > 10.0, ReadPosRankSum < −20.0. Afterward, the variants that passed were merged into one VCF per sample. Consensus sequences were generated by a custom Python script that encoded heterozygous positions as IUPAC-ambiguous bases and deletions and filtered variants as ‘N’s. 

The genomic data were partitioned along chromosomes. Phylogenetic trees were inferred with IQ-TREE v1.6.12 [41,42,43,44] using its model-test for predicting the best-fitting nucleotide substitution models for each partition and predicting support with 1000 ultrafast-bootstrap (UFBoot) and approximate likelihood-ratio test (SH-aLRT). The individual analysis parameters are available in Supplementary Data 3. The phylogenetic trees were visualized in R [45] using ggtree [46], phangorn [47], and ape [48] packages.

The genomes of 182 *C. albicans* isolates (PRJNA432884) and 96 *C. glabrata* isolates (SRR2982714-SRR2982719, SRR5239753-SRR5239784, SRR5459192-SRR5459193, SRR7609359-SRR7609360, SRR8068012-SRR8068062, SRR8241569-SRR8241571) were downloaded from the European Nucleotide Archives and processed in the same manner as described above to create a global phylogeny.

The mixed sequence type (ST) clades on the *C. glabrata* global phylogenetic tree were analyzed with the Mann–Whitney U test for difference between the inter-ST distances and the intra-ST distances.

### 2.5. Multilocus Sequence Typing

In silico MLST was performed by using the Center for Genomic Epidemiology MLST v2.0.4 [49] on the *C. glabrata* consensus samples as input. The genotypes and ST definitions [50] were downloaded from the *C. glabrata* MLST website on pubmlst.org [51] on 2020-04-30.

## 3. Results

Of the 51 yeast isolates, 30 were found to be *C. albicans*, 8 *C. glabrata*, 7 *Candida krusei*, 3 *Candida dubliniensis*, 2 *Candida tropicalis*, and 1 *Candida guilliermondii*. Only the *C. albicans* and *C. glabrata* genomes were analyzed further.

### 3.1. Antifungal Susceptibility Testing

**Figure 1:**
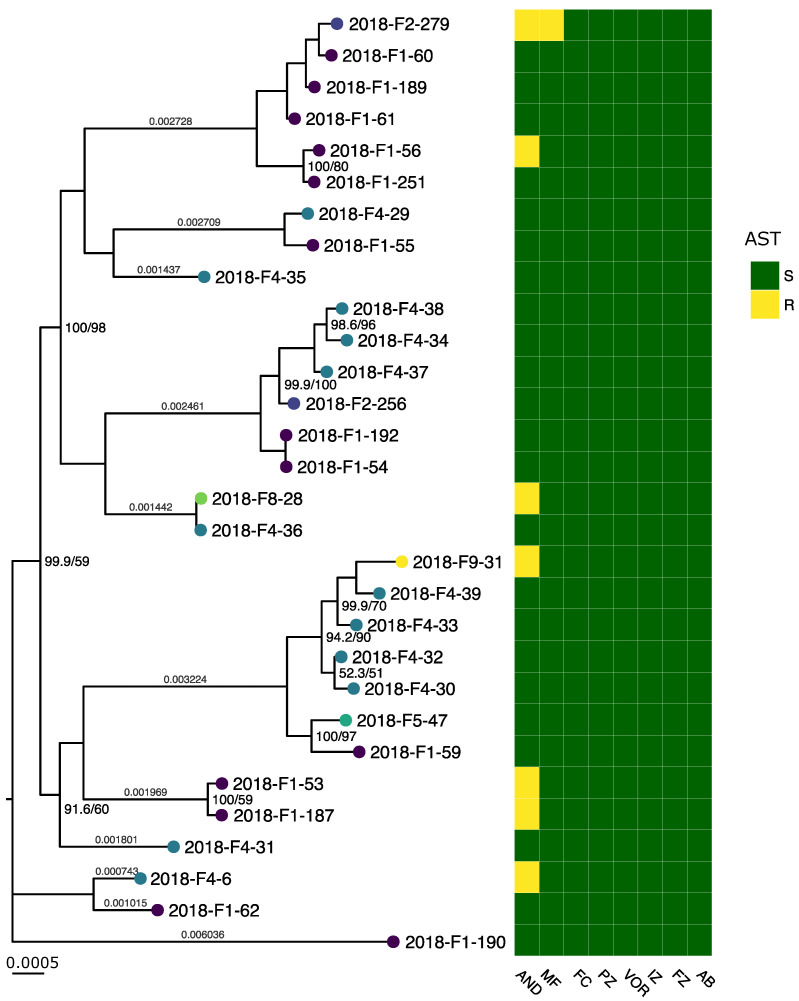
Maximum likelihood phylogenetic tree of *C. albicans* isolates using the reference sequence *C. albicans* SC5314, rooted on midpoint and displaying UFBoot/SH-aLRT support for splits with less than 100% support. The scale bar is 0.0005 substitutions per site. Tips are colored according to the DCM codes. The heatmap displays the AST results against anidulafungin (AND), micafungin (MF), 5-Flucytosine (FC), posaconazole (PZ), voriconazole (VOR), itraconazole (IZ), fluconazole (FZ), amphotericin B (AB). R corresponds to clinically resistant or NWT isolates; S corresponds to clinically susceptible or WT isolates.

**Figure 2:**
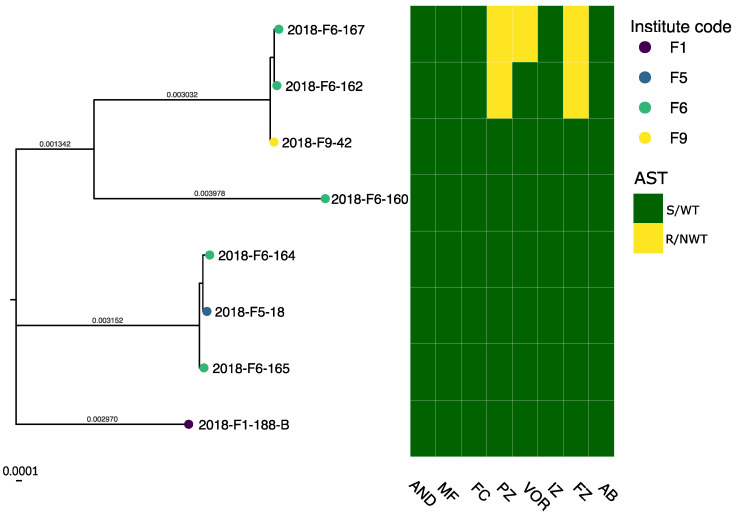
Maximum likelihood phylogenetic tree of *C. glabrata* isolates using the reference sequence *C. glabrata* CBS138, rooted on midpoint. The scale bar is 0.0001 substitutions per site. Tips are colored according to the DCM codes. The heatmap displays the antifungal susceptibility testing results against anidulafungin (AND), micafungin (MF), 5-Flucytosine (FC), posaconazole (PZ), voriconazole (VOR), itraconazole (IZ), fluconazole (FZ), amphotericin B (AB). R corresponds to clinically resistant or NWT isolates, S corresponds to clinically susceptible or WT isolates.

AST showed high susceptibility towards azoles among the *C. albicans* isolates (Figure 1); all were clinically susceptible or presented a wild-type (WT) phenotype (MIC values equal or below the ECOFF) to fluconazole, itraconazole, voriconazole, and posaconazole. The *C. glabrata* isolates (Figure 2) had lowered susceptibility to azoles. Two were resistant to fluconazole and simultaneously presented a non-wild type (NWT) phenotype (MIC values above the ECOFF) for posaconazole, with one of those isolates also presenting a non-wild type phenotype for voriconazole. All eight *C. glabrata* isolates had lower MIC values than the ECOFF for itraconazole. Furthermore, all isolates from both species were susceptible to amphotericin B. Seven *C. albicans* isolates showed increased MIC to echinocandins when compared with the respective EUCAST clinical breakpoints: all seven appeared resistant to anidulafungin, and one was also resistant to micafungin. All of the *C. glabrata* isolates were susceptible to echinocandins. The MIC values and breakpoints used for each antifungal agent can be found in Supplementary Data 2.

### 3.2. Phylogenetics and MLST

Phylogeny of the Danish *C. albicans* isolates identified three clusters with more than five isolates each, and the remaining isolates as clusters of two or singletons. The *C. glabrata* phylogeny was comprised of two clusters with three isolates each and two singletons. All larger clusters encompassed isolates from different DCMs and sources (Figures S1 and S2). Site substitution rates on branches did not indicate that any isolate was closely related to another in either phylogeny. 

MLST of *C. glabrata* isolates (Table S1) predicted five STs, with ST3 for three and ST6 for two isolates.

**Figure 3:**
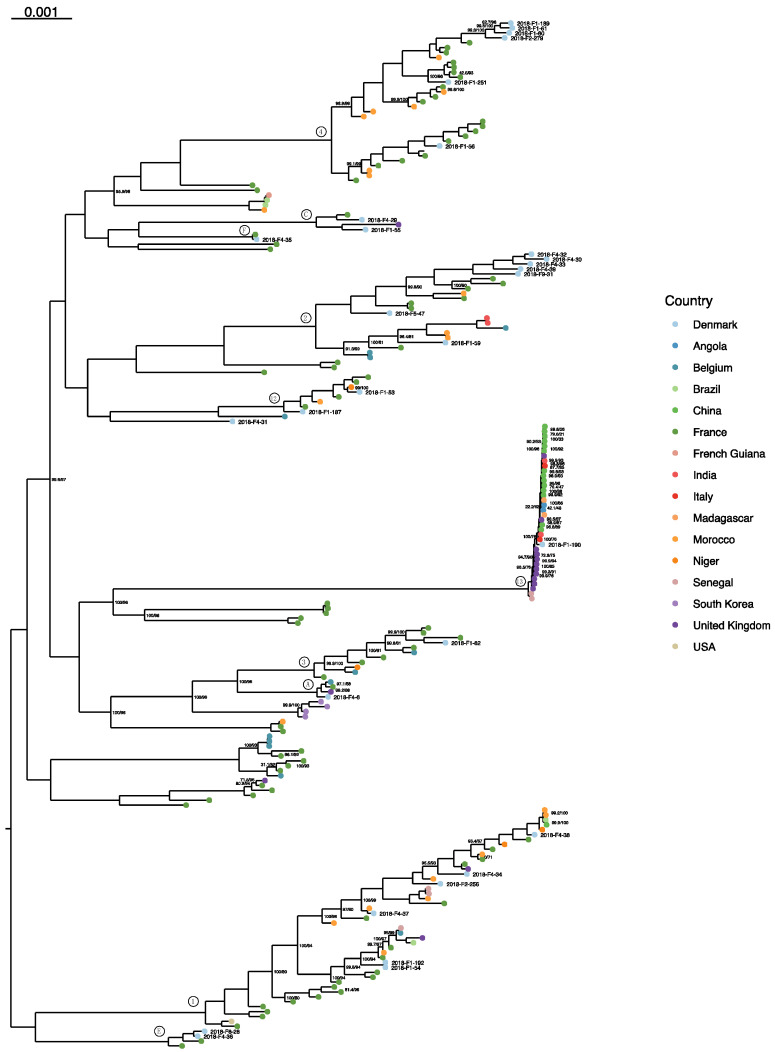
Global maximum likelihood phylogeny of *C. albicans* isolates, with the Danish isolates labeled. Tips are colored by country, the UFBoot/SH-aLRT supports are shown where lower than 100%, and the scale bar is 0.001 substitutions per site. Clades were labeled after Ropars et al. [22].

**Figure 4:**
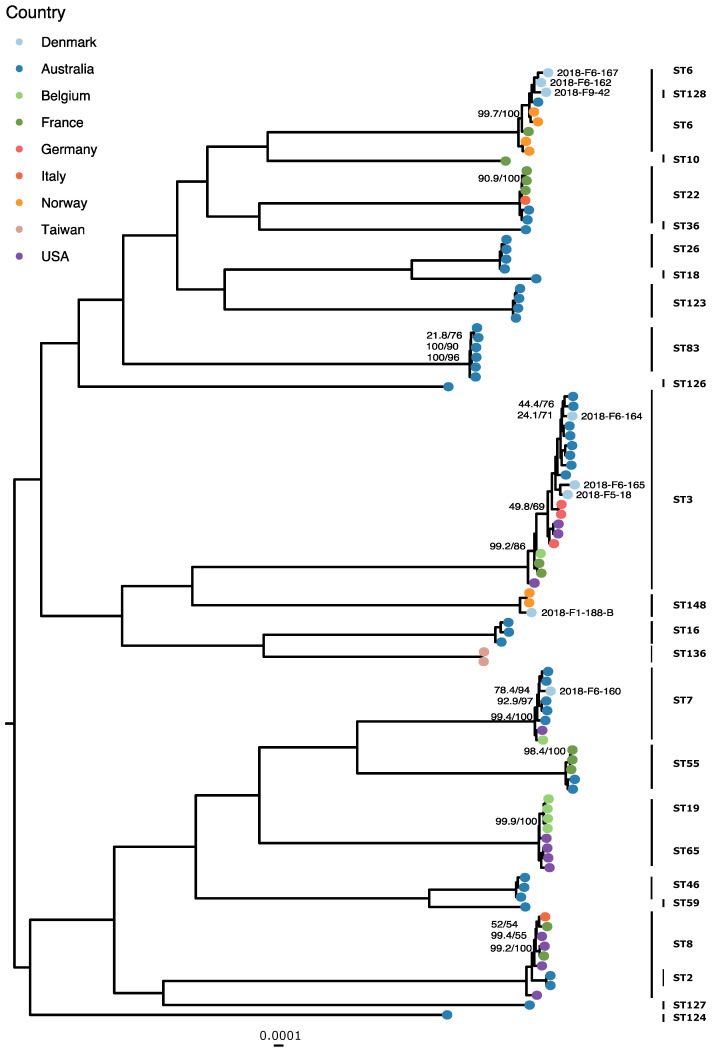
Global maximum likelihood phylogeny of *C. glabrata* isolates, with the Danish isolates labeled. Tips are colored by country, the UFBoot/SH-aLRT supports are shown where lower than 100%, and the scale bar is 0.0001 substitutions per site.

In the *C. albicans* global phylogeny (Figure 3), the Danish isolates fit into already established MLST-based clades [19,20,22]: seven isolates were in clade 2, six each in clades 4 and 1, two in clade 12, and one each in clades 13 and 3. One isolate was an out-group to clade 12. Other isolates were clustered in novel clusters suggested by Ropars et al. [22], namely, in clusters A, C, E, and F. The *C. glabrata* isolates (Figure 4) separate along the predicted STs, except for 2018-F9-42, which is placed on a clade with the ST6 samples. There are three instances of clades containing isolates from two STs: ST6 and ST128, ST19 and ST65, and ST2 and ST8. The intra-ST distances do not differ significantly from the inter-ST distances for either of these clades when tested with the Mann–Whitney U test (*p* values 0.09073, 0.4228, and 0.4081, respectively).

## 4. Discussion

In this study, we sequenced clinical *Candida* isolates collected during one day in Denmark, from which the majority of the isolates were typed to be *C. albicans* (*n* = 30), followed by *C. glabrata* (*n* = 8). These results are concordant with the findings of the incidence-based fungemia surveillance from Denmark [4] that reported an increased prevalence of *C. glabrata* candidemia in the country, although only a limited number of our isolates were isolated from blood. However, studies have shown that invasive candidiasis is frequently caused by commensal strains [25]; therefore, less-severe opportunistic infections could mirror the prevalence of candidemia.

AST showed that all *C. albicans* isolates were susceptible to azoles. Approximately 25% of those isolates (7/30) appeared to be resistant to anidulafungin, and one was also resistant to micafungin, albeit with MIC values only one- or two-fold dilutions above the EUCAST breakpoint. This would be a concerning development, as in the 2012–2018 period, no anidulafungin-resistant *C. albicans* isolates have been detected in Denmark [2]. Furthermore, echinocandin-resistant *C. albicans* remain rare worldwide [9,52,53], with few cases reported and mostly corresponding to a decrease in susceptibility following long-term treatments with these antifungal agents [54,55,56,57,58,59]. However, we propose that insufficient standardization of the AST method used is responsible for obtaining the observed higher MIC values, as demonstrated by the classification of these isolates as clinically susceptible to echinocandins when the Clinical and Laboratory Standards Institute (CLSI) breakpoints are applied instead (S ≤ 2 mg/L) (personal communication). The *C. glabrata* isolates were all susceptible to echinocandins by both interpretations, in contrast to the global observations showing a higher prevalence of echinocandin resistance in *C. glabrata* than *C. albicans* [60,61]. However, the low number of *C. glabrata* isolates tested might not accurately reflect the effective *Candida* epidemiology in the country. Of the *C. glabrata* isolates, 25% (2/8) presented an NWT or resistant phenotype for certain azoles. This is in accordance with the results of the national surveillance reporting an increased number of NWT *C. glabrata* samples. [2,4]

The resistant or NWT isolates, with the exception of two pairs of isolates, did not cluster phylogenetically (Figure 1 and Figure 2), and we could assume that the genetic mechanisms conferring resistance were acquired independently. While isolates 2018-F1-53 and 2018-F1-187 were clustered together and both exhibited increased MIC values to anidulafungin, it has been reported that *C. albicans* rapidly acquires resistance-conferring mutations in the gene coding for the drug target [62]. Similarly, 2018-F1-162 and 2018-F1-167 clustered together and presented an NWT for azoles, but *C. glabrata* is also known for developing resistance during treatment [62,63]. In both cases, further analysis would be required to investigate the genetic background of the observed phenotypes.

The majority of the *C. albicans* isolates (19/30) were from the most-sampled global clades, namely clades 2, 1, and 4 (Figure 3). The within-clade diversity was considerable in these clades, although the inter-clade distance was higher. The Danish isolates were scattered in clade 1, and only 2018-F1-54 and 2018-F1-192 were clustered together, separating from a cluster of French isolates. In clades 2 and 4, the Danish isolates are separated into two clusters each, and the larger clusters only contain Danish isolates. Although the population is under-sampled, geographically constrained clonal expansion could explain the observed phenomenon. The remaining 11 isolates were placed into three MLST-established and four putative clades, and one remained a singleton. One of these isolates was embedded in clade 13 that contained strains adapted to the genitalia, with a very low degree of polymorphism and heterozygosity, characteristics that 2018-F1-190 also shared. 

The *C. glabrata* isolates were largely separated along ST types in the global phylogeny (Figure 4), and the intra-cluster diversity was lower than what could be observed in the case of *C. albicans*. *C. glabrata* is haploid; therefore, heterozygosity does not contribute to the genetic diversity of the species. Approximately 63% (*n* = 5) of the isolates belonged to the globally prevalent ST3 and ST6. There were three instances of isolates of different STs intermixing within clades, probably due to genetic drift affecting one of the six loci in the MLST scheme resulting in a different ST designation, despite the inter-ST genetic distances being comparable to the intra-ST genetic distances. 

In summary, we whole-genome sequenced Danish *Candida* isolates to uncover the population structure. We found that the majority of the isolates belonged to clades that were widespread in the world, despite some evidence of local clonal expansion. This study suggests that both *C. albicans* and *C. glabrata* are truly globally distributed, opportunistic pathogens without geographical restrictions on diversity.

## Data Availability

The data presented in this study are available in the Supplementary Materials. The whole-genome-sequencing data generated during the project were deposited into the European Nucleotide Archive under the project number PRJEB40738.

## Supplementary Materials

The following are available online at https://www.mdpi.com/article/10.3390/jof7110962/s1: Supplementary Material, Supplementary Data 1: Sample metadata; Supplementary Data 2: MIC and breakpoints used; Supplementary Data 3: IQ-tree outputs with analysis parameters.
