# Supplementary material for: Danish Whole-Genome-Sequenced Candida albicans and Candida glabrata Samples Fit into Globally Prevalent Clades
Source: J Fungi (Basel). 2021 Nov 12;7(11):962. doi: 10.3390/jof7110962 (PMC8622182; doi:10.3390/jof7110962)
Supplement: Supplementary file 1 [file jof-07-00962-s001.zip › Supplementary_material.pdf]

Supplementary material

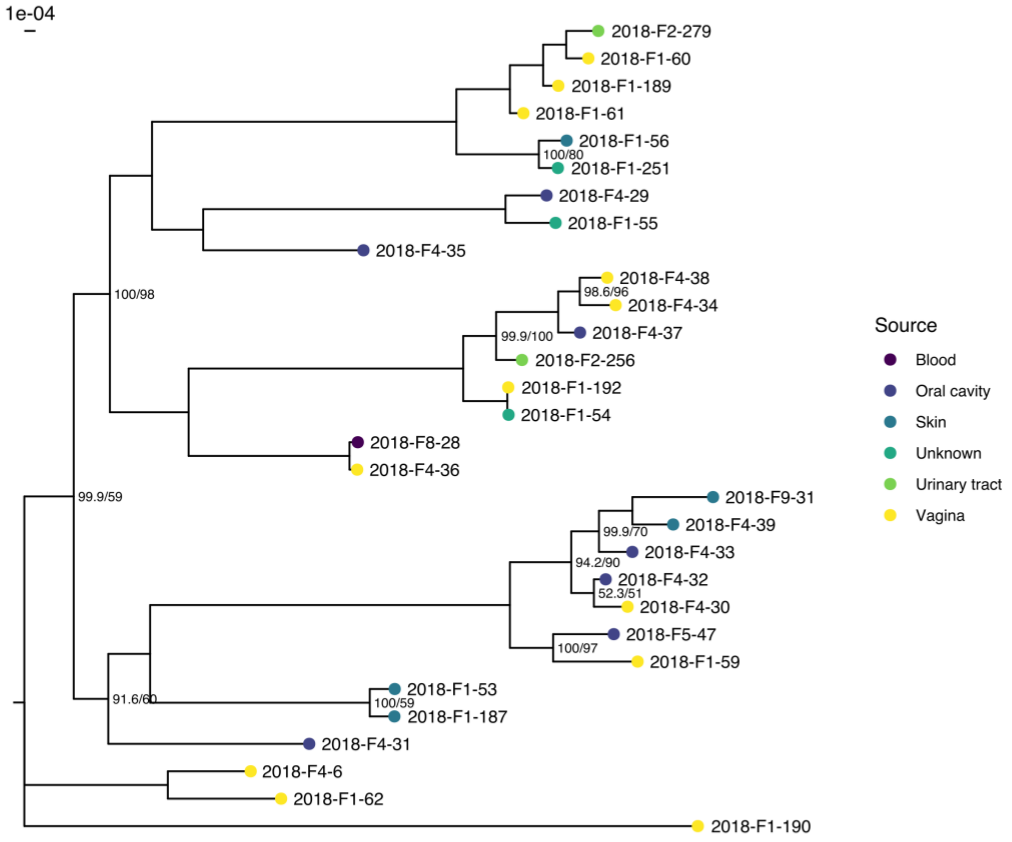

Figure S1. Maximum likelihood phylogenetic tree of *Candida albicans* isolates using the reference sequence *Candida albicans* SC5314, rooted on midpoint and displaying UFBoot/SH-aLRT support for splits with less than 100% support. Tips are colored according to the standardized sample sources.

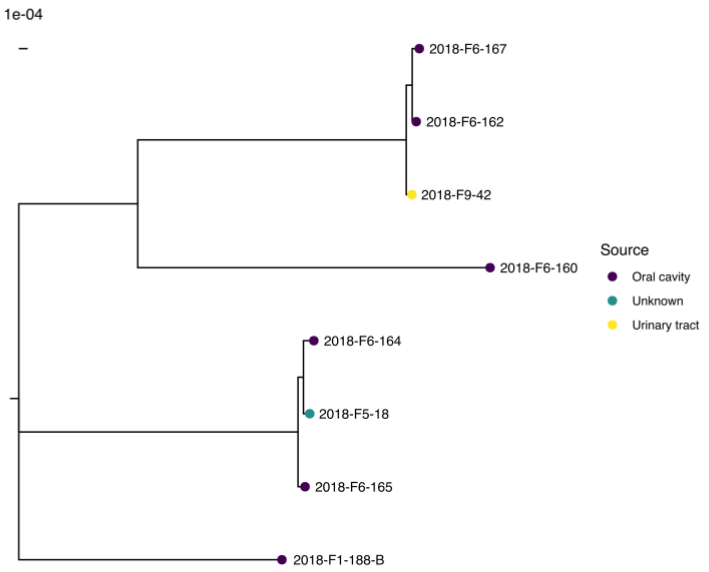

Figure S2. Maximum likelihood phylogenetic tree of *Candida glabrata* isolates using the reference sequence *Candida glabrata* CBS138, rooted on midpoint. Tips are colored according to the standardized sample sources.

Table S1. In silico multilocus sequence typing of *C. glabrata* isolates predict the samples to belong to five sequence types.

Sample	ST
2018-F1-188-B	148
2018-F5-18	3
2018-F6-160	7
2018-F6-162	6
2018-F6-164	3
2018-F6-165	3
2018-F6-167	6
2018-F9-42	128
